# A Cross-cultural Perspective on Intrathecal Opioid Therapy Between German and Iranian Patients

**DOI:** 10.1007/s11013-020-09682-6

**Published:** 2020-07-28

**Authors:** Barbara Kleinmann, Nayereh Khodashenas Firoozabadi, Tilman Wolter

**Affiliations:** 1 Interdisciplinary Pain Center, Medical Center-University of Freiburg, Faculty of Medicine, University of Freiburg, Breisacherstr. 64, 79106 Freiburg, Germany; 2 Pain Clinic, Department of Anesthesiology, Khatam ol Anbia Hospital, Tehran, Islamic Republic of Iran

**Keywords:** Intrathecal opioid therapy, Long-term therapy, Unwanted side effects, Cross-cultural factors

## Abstract

Patients often adhere to intrathecal opioid therapy (IOT) for many years, despite the lack of scientific evidence for its efficacy and the scarce knowledge about long-term effects. Moreover, there is no knowledge on how the efficacy of IOT is influenced by cultural factors. We assessed the long-term efficacy and frequency of side effects of IOT in two culturally different patient samples. A chart review was conducted of all patients with IOT, who had been treated in interdisciplinary pain centers in Freiburg and in Tehran in a 15-year span. Personal data, diagnosis, duration of pain disease, pump type in use, revision operations, and opioid doses were recorded. Patients completed a questionnaire containing pain scores, pain-related disability (PDI), anxiety, depression, and unwanted side effects. Fourteen Iranian and 36 German patients (32 m/18 f) were studied. Mean duration of IOT was 10.2 years. Pain levels prior to IOT were 7.64 (NRS) (range 4–10, SD 1.64), 3.86 (range 0–9, SD 2.32) directly after pump implantation, and 4.17 (range 0–10, SD 2.11) at time of follow-up. Iranian patients had significantly lower pain levels directly after implantation, depression scores, and pain-related disability. Frequent side effects were obstipation, sexual dysfunction, urinary retention, and fatigue. Most side effects were significantly less frequent in the Iranian sample. There were no severe complications or permanent neurological deficit. Our study demonstrates the effectiveness of IOT also for long-term application. Differences in clinical efficacy are partially due to cultural factors. Side effects are frequent but not limiting patient satisfaction.

## Introduction

Intrathecal opioid therapy (IOT) has been used for over 40 years in the treatment of malignant and non-malignant chronic pain. There have been numerous reports on beneficial outcomes after intrathecal pump implantation (Paice, Penn, and Shott 1996; Hassenbusch et al. 2004; Smith et al. 2008). However, there is still no clear scientific evidence to support the use of intrathecal opioid pumps also in non-malignant pain, as Harden pointed out in a recent editorial (Harden, Argoff, and Williams 2014). Moreover, there is the impression that the implantation rates in Western countries are stagnating (Levy 2012). One reason for this could be a growing awareness of unwanted side effects of IOT, such as granuloma formation or hormonal changes (Duarte et al. 2013) in the course of the therapy. On the other hand, particularly in emerging countries in Asia, the frequency of intrathecal opioid therapy still seems to be growing (Transparency-Market-Research 2014). This raises the question whether or not efficacy of IOT is influenced by cultural aspects. Culture in this context may be considered as a shared system of values, beliefs, and learned patterns of behavior (Bigby 2003).

We sought to enlarge the data base of a recent study on 36 patients who had been treated in Germany (Kleinmann and Wolter 2017), performing the same data collection on Iranian patients. We hypothesized that the pain relief, the level of satisfaction elicited by IOT, and possibly also the rate of unwanted side effects would be the same in both samples.

## Materials and methods

### Patients

**Fig. 1:**
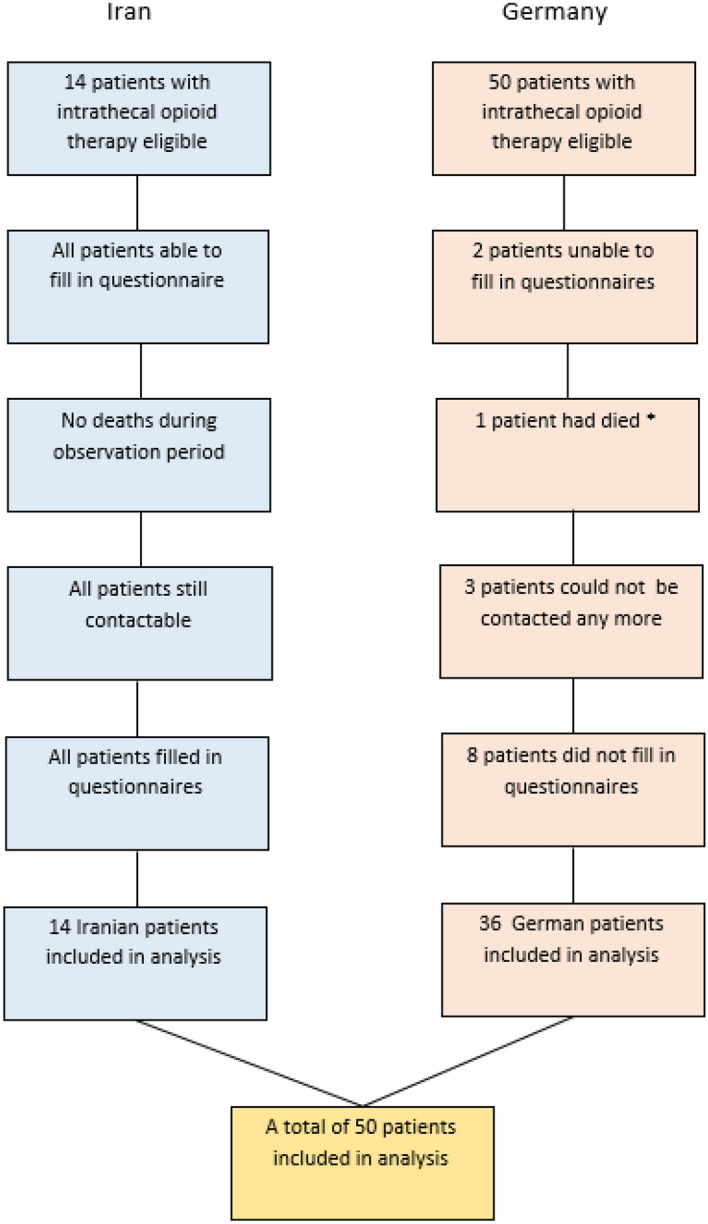
Patients eligible and patients analyzed, *From reason independent from intrathecal opioid therapy

The retrospective study was approved by the Institutional Review Boards of both institutions. (Medical Ethics Committee Shefa Neuroscience Research Center, Code No.: 983014, and Ethic Committee of the University Hospital Freiburg, No.:196/15). All patients with intrathecal opioid pumps, who had been treated at our institutions between 01.01.1990 and 31.12.2015 and who had carried an implanted intrathecal opioid pump for at least 3 years, were eligible (in Germany). The Iranian part of the study included patients who had been operated within the last 3 years, thus between 01.01.2013 and 31.12.2015. In the German population during that time span, no new patients were eligible, so that this had no influence on patient selection. While in the Iranian part of the study all patients eligible completed the questionnaire, in the German part some patients refused or were unable to participate in the study. Overall, 50 patients completed the questionnaire (Fig. 1).

In both samples, the indication for implantation of an intrathecal opioid pump was based on the following criteria:

- Intractable chronic pain.
- Insufficient or missing pain reduction under high dose oral/transdermal opioids.
- Psychological exam ruling out major psychiatric comorbidity.

### Chart Review

Information regarding age, gender, diagnosis, age at time of implant, duration of disease, pump type in use, revision operations (scheduled and unscheduled), opioid doses, drug holidays, and spinal catheter position was recorded from the charts.

### Questionnaires

A questionnaire was sent to the patients by post. The questionnaire contained the following items:

- pain scores on the Nominal Rating Scale (NRS) before and under IOT,
- whether the actual pain was the same as the pain prior to pump implantation,
- effects of the IOT on sleep and on mobility,
- typical unwanted side effects of opioid therapy, and whether these were present currently or in the past,
- satisfaction with therapy and whether the operation would be considered again,
- pain-related disability and
- anxiety and depression.

### Statistical Analysis

A computer software package (GraphPad Prism, Version 5.01, GraphPad Software, Inc. La Jolla, USA) was used to conduct statistical analyses. Initially, descriptive statistics were applied to all measures. In case of normal distribution, paired or unpaired t tests were employed to calculate differences between groups. The Wilcoxon matched pairs test was used to calculate the statistical significance of the differences in mean NRS scores and for anxiety/depression scores. The Mann–Whitney *U* test was used to compare mean pain/anxiety/depression ratings in different groups. *p* < 0.05 was considered statistically significant. For normally distributed measures Pearson correlations and for not normally distributed measures Spearman correlations were calculated.

### Sample Size Estimation

With α = 0.05, a power of 0.8 and given a standard deviation of 1.6 on the NRS in the pain scales and a detectable alternative of 1.6 points on the NRS the sample size for the Mann–Whitney *U* test was estimated to be 13.

With α = 0.05, a power of 0.8 and given a standard deviation of 4.26 points for depression and 4.20 points for anxiety on the HADS D scale and a detectable alternative of 4.2 points on the HADS D scale for the Mann–Whitney *U* test was estimated to be 13.

## Results

### Patients

Fifty patients (32 m/18 f) completed the questionnaire. Mean age at time of the study was 60.3 years (range 30–84 years, SD 11.3 years). Mean duration of IOT at the time of filling in the questionnaire was 10.2 years, (range 1.5–24 years, SD 5.3 years). There were statistically significant differences between the Iranian and the German patients regarding age and duration of intrathecal therapy (Table 1).

**Table 1:** Patient characteristics, diagnoses, and details of intrathecal opioid application

Item	Iran	Germany	Total	p
n	14	36	50	n.a.
M/f	11 m/3 f	21 m/15 f	32 m/18 f	0.2108*
Age/years				0.0048**
Mean	53.7	62.9	60.3	
Range	42–77	30–84	30–84	
SD	9.6	11.0	11.3	
Time since pump implant/years				0.0004
Mean	6.1	11.8	10.2	
Range	1.5–13.0	3.7–24.0	1.5–24.0	
SD	4.4	4.8	5.3	
Diagnoses/n				n.a.
CLBP		29	29	
SCI	9	1	10	
RLS		1	1	
PLP	2	3	5	
Spine fracture	1	1	2	
Sacral cycts		1	1	
CRPS	1		1	
Abdominal pain	1		1	
Pumps/n				1.0000*
Gas driven	12	32	44	
Programmable	2	4	6	
Opioids				
Morphine				0.0224***
n patients	10	30	40	
Mean dose****	2.47	4.61	4.06	
Buprenorphine				0.0571***
n patients	3	4	7	
Mean dose****	0.087	0.150	0.126	
Hydromorphone				n.a.
n patients		1	1	
Fentanyl				n.a.
n patients	1	1	2	

*Unpaired t test, **Mann–Whitney test, ***Fishers exact test, **** mg/die, n.a. = not applicable

### Diagnoses

Diagnoses were different between Iranian and German patients. While among the Iranian patients 11 had spinal cord injury leading to incomplete or complete paraparesis, 29 of the 36 German patients had chronic low back pain. 23 of these patients had spine operations in their medical history and 14 of these patients had spondylodesis. Three German patients had phantom limb pain and one patient each had a brachial plexus avulsion, cervicobrachialgia secondary to spinal cord injury, severe restless legs syndrome, pain due to thoracic fracture, and multiple sacral cysts (Tarlov cysts).

### Intrathecal Pump Systems

Forty-four patients had gas-driven pumps and six patients had programmable pumps. Thirty-six patients still carried their first implanted pump. Eight patients had one pump exchange and six patients had two pump exchanges.

### Opioid Doses

The mean dose in those patients receiving morphine sulfate was 4.06 mg/day. In the patients who had buprenorphine, the mean dose was 0.126 mg/day (Table 2). German patients had significantly higher morphine doses than Iranian patients (Table 1).

**Table 2:** Comparison of outcome parameters

Item	Iran	Germany	Total	p
n	14	36	50	
Pain/NRS				
Before treatment				
Mean	6.86	7.98	7.64	0.0309*
Maximal	9.07	9.09	9.08	0.9612*
Minimal	5.07	6.82	6.28	0.0188*
Directly after pump implant				
Mean	1.71	4.87	3.86	< 0.0001*
Maximal	2.79	5.75	4.88	< 0.0001*
Minimal	1.00	4.48	3.37	< 0.0001*
Last two weeks before follow-up				
Mean	3.57	4.44	4.17	0.2040*
Maximal	4.86	5.28	3.37	0.5774*
Minimal	2.36	3.81	5.16	0.0301*
Change in % (SD)				
Before treatment-directly after pump implant	74.40 (24.47)	40.67 (26.89)	50.51 (30.22)	0.0002*
Before treatment-last 2 weeks before follow-up	46.84 (32.26)	44.30 (29.94)	45.04 (31.31)	0.7949*
HADS A	5.07	6.78	6.30	0.1997*
HADS D	3.57	7.22	6.20	0.0057*
HADS total	8.64	14.00	12.50	0.0306*
PDI				
Familiar and domestic duties	4.64	6.11	5.70	0.3273**
Recreation	2.93	6.04	5.14	0.0027**
Social activities	4.50	5.27	5.05	0.5110**
Profession	2.64	6.15	5.11	0.0047**
Sexual life	7.14	6.60	6.77	0.2002**
Vitally indispensable activities	1.57	3.65	3.07	0.0071**
Self-care	0.57	2.96	2.26	0.0002**
PDI total	24.00	34.71	31.71	0.0661**

Bold: *p* < 0.05, *NRS* numeric rating scale, *HADS* Hospital Anxiety and Depression Scale, *PDI* Pain Disability Index, *PDI*: 0 no disability, 10 maximal disability, *Unpaired t test, **Mann–Whitney test

In patients receiving morphine, there was no correlation between the opioid dose and pain reduction directly after implantation (Pearson *r* (39) = 0.1486, *p* = 0.3871), but pain reduction at time of follow-up showed an inverse correlation to the opioid dose (Pearson *r* (39) = − 0.3420, *p* = 0.0412).

### Outcome

**Fig. 2:**
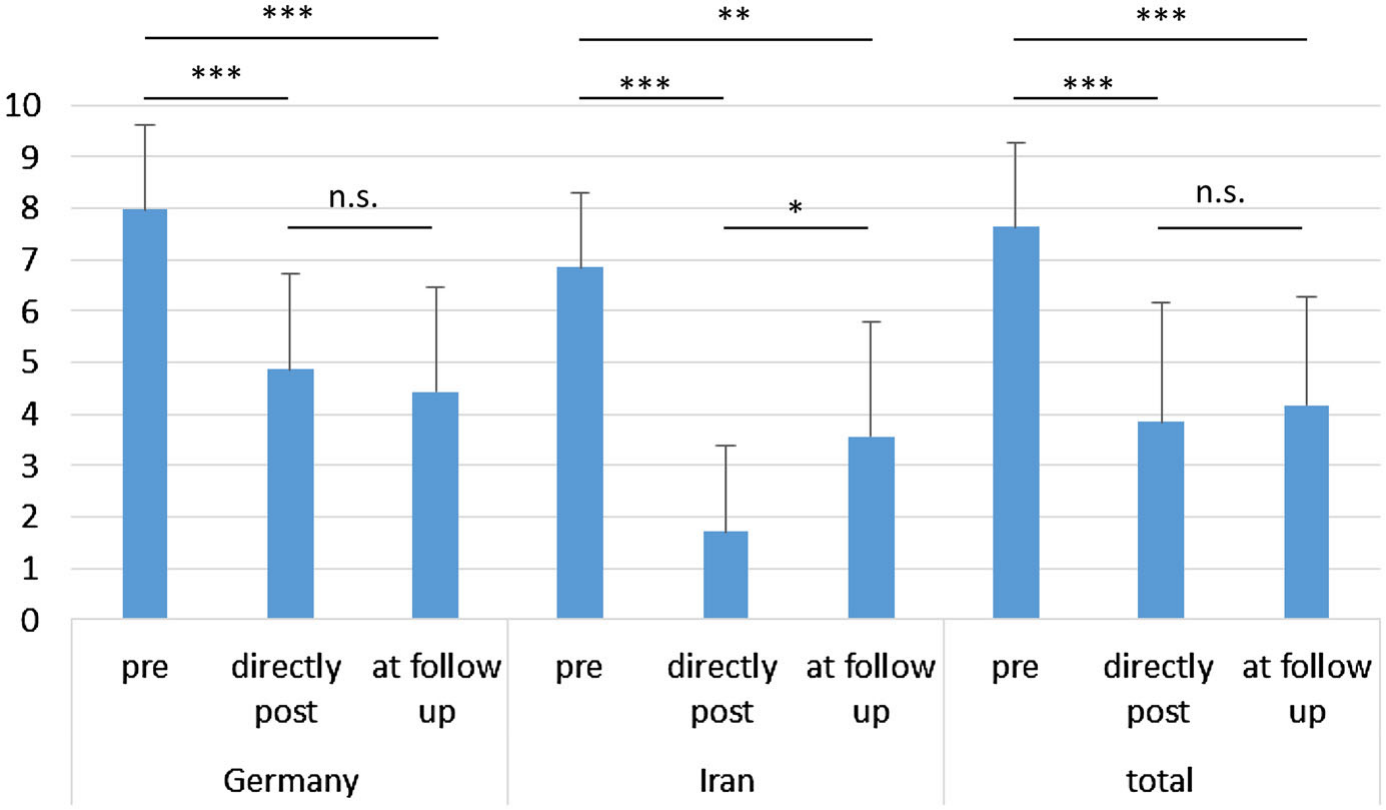
Mean pain on the NRS before pump implantation (pre), directly after pump implantation (directly post) and at follow-up (paired *t* test), *p = 0.0005, **p = 0.0013, ***p < 0.0001, n.s. = not significant, error bars: SD

Overall, mean pain levels prior to pump implantation were 7.64 (NRS) (range 4–10, SD 1.64). Mean pain levels directly after the pump implantation were 3.86 (range 0–9, SD 2.32) and at time of follow-up 4.17 (range 0–10, SD 2.11) (Table 2, Fig. 2). The difference between the pre-and post-implantation values was statistically significant (all *p* > 0.0001) while there were no statistically significant differences between the pain levels at the two post-implant time points. Iranian patients had lower pain levels pre- and directly post-implantation. At the time of follow-up, mean pain levels in the Iranian patients were still lower than in the German patients, but this difference was statistically significant only for the minimal pain level. The mean percentage of pain reduction was also higher in the Iranian sample. However, this difference was only statistically significant directly after implantation, but not at the time of follow-up.

### Anxiety/Depression

Overall, at the time of follow-up, mean values for depression were 6.20 (SD 4.30), for anxiety 6.30 (SD 4.20) and total anxiety and depression values 12.50 (SD 7.94). Iranian patients had significantly lower depression scores than German patients, but there was no statistically significant difference between the groups in anxiety scores. Overall, depression levels correlated with the mean pain levels directly post-implantation and at time of follow-up (*r* (46) = 0.4782, *p* = 0.0010, *r* (46) = 0.4088, *p* = 0.0048), but not with preoperative mean pain levels. Anxiety levels correlated only with mean post-operative pain levels (*r* (44) = 0.3487, *p* = 0.0204), but not with pain levels preoperatively or at time of follow-up.

### Sleep and Mobility

Fourteen patients rated their sleep as significantly improved and 23 somewhat improved. In nine patients, it was unchanged and in four patients it was significantly worse than before pump implantation. Mobility was subjectively rated as significantly improved by nine patients and somewhat improved by 22 patients. Eleven patients rated their degree of mobility unchanged, while it was somewhat worsened in four patients and significantly worsened in three patients.

### Pain Disability Index (PDI)

Overall mean PDI scores ranged from 1.57 (SD 2.57, item ‘self-care’) to 6.77 (SD 3.38, item ‘sexual life’). The mean total PDI score was 24.00 (SD 16.48, range 0–45). There were statistically significant differences between the German and the Iranian patients regarding the items recreation, profession, vitally indispensable activities and self-care (Table 2).

### Complications and Operative Revisions

Twenty-two patients had unscheduled surgery. In 17 cases, catheter revisions due to dislocation, leak or occlusion were carried out. Two patients needed a relocation of the pump due to pain at the pocket site, one revision of the connector was necessary due to skin atrophy and in two cases pump removal due to infection and later re-implantation was carried out.

### Side Effects

**Fig. 3:**
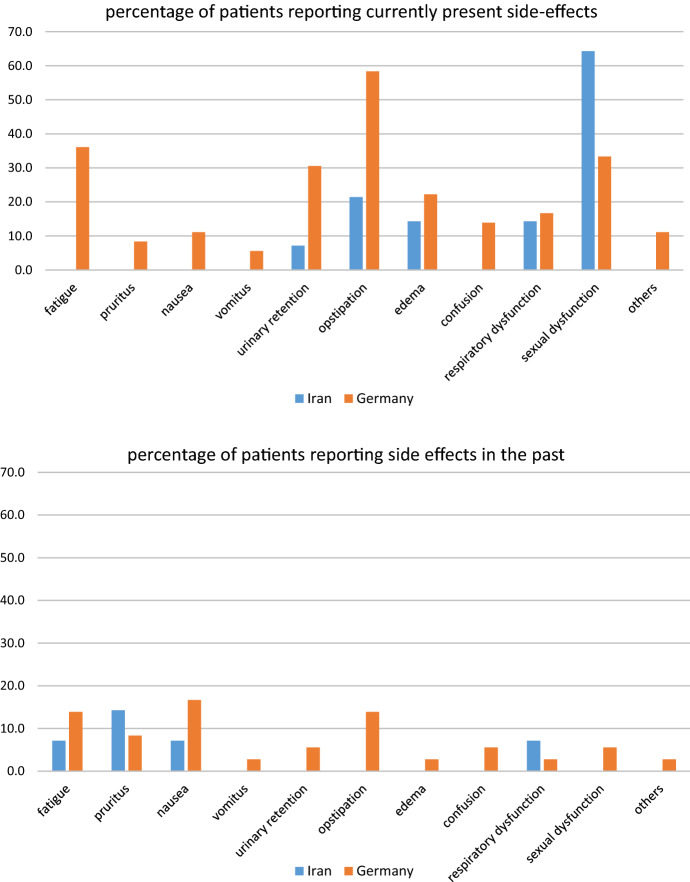
Percentage of patients reporting currently present side effects (a) or side effects occurring in the past during intrathecal opioid therapy

The most common unwanted side effects reported by the patients were fatigue, obstipation, urinary retention, and sexual dysfunction. The various percentages of side effects which were currently present or which the patients had encountered in the course of therapy with intrathecal opioids are displayed in Fig. 3 and. In the Iranian patients, most side effects were less frequently present than in the German patients or they were even completely absent. In some of these instances, the data did not allow comparisons by mean of Fisher’s exact tests and in the remaining instances the difference was statistically significant (Table 3).

**Table 3:** Comparison of number of unwanted side effects

Item	Iran	Germany	Total	p*
n	14	36	50	
Fatigue				0.0148
Currently present	0	13	13	
Never present	1	5	6	
Earlier present	13	18	31	
Pruritus				n/a
Currently present	0	3	3	
Never present	12	3	15	
Earlier present	2	30	32	
Nausea				< 0.0001
Currently present	0	4	4	
Never present	13	6	19	
Earlier present	1	26	27	
Vomitus				n/a
Currently present	0	2	2	
Never present	14	33	47	
Earlier present	0	1	1	
Urinary retention				n/a
Currently present	1	11	12	
Never present	13	23	36	
Earlier present	0	2	2	
Obstipation				0.0041
Currently present	3	21	24	
Never present	11	10	21	
Earlier present	0	5	5	
Edema				n/a
Currently present	2	8	10	
Never present	12	27	39	
Earlier present	0	1	1	
Confusion				
Currently present	0	5	5	n/a
Never present	14	29	43	
Earlier present	0	2	2	
Respiratory dysfunction				n/a
Currently present	2	6	8	
Never present	11	29	40	
Earlier present	1	1	2	
Sexual dysfunction				n/a
Currently present	9	12	21	
Never present	5	22	27	
Earlier present	0	2	2	
Others				n/a
Currently present	0	4	4	
Never present	14	31	45	
Earlier present	0	1	1	

*Chi square test

*n/a* not applicable

### Treatment Satisfaction

Twenty-two patients reported being very satisfied with IOT, 25 reported being fairly satisfied, one patient was undecided and two patients were rather unsatisfied with therapy. Forty-five patients would undergo the pump implantation again, while four patients would not do so. There were no differences between the degree of treatment satisfaction in the Iranian and the German patients.

### Oral Medication

Seven patients took no oral pain medication. 21 patients took one compound, 12 patients took two compounds, eight patients took three compounds and two patients took more than three different analgesics.

Twenty patients took NSAIDs, ten patients were taking strong opioids and two patients took weak opioids, 12 patients were on antidepressants, 18 patients on anticonvulsants, four patients on muscle relaxants and nine patients took other drugs.

Iranian patients took no strong opioids and weak opioids only in one case. They took anticonvulsants and antidepressants more frequently than German patients (Table 4).

**Table 4:** Distribution of concomitant oral medication in the two samples

Type of medication	n/Iran	%/Iran	n/Germany	%/Germany	n/total	%/total
NSAID	0	0.0	20	55.6	20	40.0
Weak opiods	1	7.1	1	2.8	2	4.0
Strong opiods	0	0.0	10	27.8	10	20.0
Muscle relaxants	0	0.0	4	11.1	4	8.0
Anticonvulsants	11	78.6	7	19.4	18	36.0
Antidepressants	7	50.0	5	13.9	12	24.0
Others	6	42.9	3	8.3	9	18.0

## Discussion

The present study shows a good efficacy of intrathecal opioid therapy after long-term application. Typical side effects were frequent, but did not affect the patients` acceptance of the treatment method. Surgical complications were relatively rare, a granuloma formation was not observed in the two samples.

To the best of our knowledge, this is the first report on cross-cultural differences in IOT. We found that the initial pain relief elicited by IOT was stronger among the Iranian patients than among the German patients. At follow-up, there were no longer differences regarding pain reduction between the two samples. However, the Iranian patients had a significantly lower rate of side effects. Moreover, German patients had higher depression scores than the Iranian patients, but anxiety scores were not significantly higher.

What is the explanation for these differences? First, there is a striking difference in diagnoses treated. While in the Iranian sample, a large portion of spinal cord injuries was treated, low back pain, mostly secondary to multiple lumbar spine surgeries prevailed in the German sample. While Paice et al. in an early study found a slightly higher efficiency of intrathecal opioid application in patients with nociceptive pain (Paice, Penn, and Shott 1996), recent guidelines (Deer et al. 2017) report no evidence of differences in intrathecal opioid efficacy between nociceptive and neuropathic pain, based on a study by Reig and Abejon (2009). Interestingly, this study also found a difference regarding gender: while only 66% of the women had pain reduction of more than 50%, in men this percentage was 85%. Such gender difference, however, would not fully explain the net variance in initial pain reduction in our study (74.4% in the Iranian vs 40.6% in the German sample).

Further differences can be taken into account: the current opioid dose in the Iranian patients was significantly lower than in the German patients, however with a shorter follow-up. This observation, however, would be compatible with a slow dose increase over time. Interestingly, higher morphine doses correlated inversely with pain reduction in the whole sample.

For many chronic pain syndromes, however, medication alone is not sufficient. Also the psychological and social aspects of pain must be considered, as the cause of pain is often multifactorial. Psychological comorbidities and disorders can have a negative impact on pain perception. Central effects of opioids can as well affect pain as can psychological disorders such as depression and anxiety. Thus, in order to assess pain in patients with opioid intake, these factors must be taken in account (Hooten 2016; Webster 2017).

The Iranian patients had lower depression scores than the German patients, which might contribute to a better pain-relieving effect. The conjunction of pain and depression has been highlighted in the literature several times (Bair et al. 2003; McWilliams, Goodwin, and Cox 2004). Also in the present study higher post-operative pain scores correlated with higher depression scores. Interestingly, the Iranian patients more frequently took antidepressants (and anticonvulsants) as concomitant oral medication, while in the German sample NSAIDs and strong opioids were the most frequent oral medications. It could be argued that depression was treated more effectively in the Iranian patients than in the German patients, but given the limited efficacy of antidepressants (Turner et al. 2008; Kamenov et al. 2017) and the large net variance in depression scores, this argument does not seem convincing. At least for the incidence of major depressive episodes, a male/female ratio of 1:1.5–2 is known (Bromet et al. 2011). Given the higher male prevalence in the Iranian sample, this could also explain the lower depression scores in the Iranian sample. Moreover, in middle-income countries, the incidence of major depression was reported to be lower than in highly industrialized countries (Bromet et al. 2011).

Differences between the two samples were also observed regarding pain-related disability as measured by the PDI. These differences are striking, as due to the high portion of spinal cord injuries in the Iranian patients, a contrary result could probably have been expected. Although the true grade of invalidity in the Iranian patients, due to the high rate of spinal cord injuries, was probably higher than in the German patients, they rated their invalidity significantly lower.

Thus, cross-cultural differences probably also play a role. For instance, cross-cultural differences in pain patterns have been observed in patients with low back pain between Germany and England (Raspe et al. 2004) and even among different regions in Germany (Schmidt et al. 2007). Cross-cultural studies in pain medicine, particularly regarding opioid therapy, are rare. Previous cross-cultural studies regarding pain medicine and involving Iranian participants, referred to the validation of questionnaires, such as the McGill pain questionnaire (Adelmanesh et al. 2011), the Oswestry disability index (Mousavi et al. 2006) or the low back outcome score (Azimi et al. 2016).

Zarghami (2015) stated that Iran has the world’s second highest rate of severe opioid addiction (Zarghami 2015). Since the 1980s, opium addiction became more prevalent for a number of social reasons such as war, unemployment, homelessness; it is a problem irrespective of age or social and economic classes. He further states that opioid consumption in Iran has historical roots reaching back as far as to the Safavids’ empire in the 15th century, when “opium smoking was a part of recreational activities of upper social class”. Three centuries earlier, the famous Iranian poets Hafez and Saadi had already praised the use of opium. This may influence the attitude towards opioid consumption even today and induce a positive expectation regarding the pain-relieving effect. An Iranian study on intrathecal opioids including 10 veterans of the Iraq–Iran war showed a global pain relief of 60%. Not only was pain relieved, but also physical activity and sleep were improved in most of these patients (Godsi et al. 2011).

Also in Germany, there was a long history of opioid use, beginning with Theophrastus from Hohenheim (Paracelsus), who in the beginning of the fifteenth century invented Laudanum, a mixture of 90% wine and 10% opium. This tincture was very popular in the eighteenth and nineteenth century. Only from 1929 on, the free disposal of opioids was forbidden. Since that time in Germany, unlike Iran, opioids are perceived as strong medical compounds or as illegal drugs.

A number of limitations of the study have to be addressed: first, the sample size, although being relatively big compared to similar studies on IOT, is still relatively small for drawing firm comparisons between the two subgroups studied. Moreover, the differences in diagnoses, age, gender, and duration of follow-up of the patients make it difficult to attribute the findings to cross-cultural factors alone. Further, possible confounding factors, such as socioeconomic state or epigenetic aspects (Liang et al. 2015), were not assessed. The cultural factors contributing to the differences in the effects of IOT could be examined more closely by qualitative interviews with patients and their family members. Qualitative interviews would allow an in depth investigation of possible cultural differences contributing to the efficacy and/or to the side effects of intrathecal opioid therapy. As personal beliefs and opinions towards therapies such as intrathecal opioid therapy or opioid therapy in general may also underlie cultural influences, these interviews could focus on possible differences in attitudes towards implanted devices as well as towards opioid use in pain therapy. With the help of the quantitative interviews, those affected could be asked about their expectations of the therapy. An interesting question would also be, which role their pain and the related restrictions play for the family and their role in society. This would also allow to draw conclusions about the therapy results and the different dosages or medication required.

The strengths of the study are the overall long follow-up of over 10 years and the high return rate of 78%.

The present study demonstrates the long-term efficacy of IOT in two clinically and culturally different populations. As most of the research on IOT has been conducted in industrialized western countries up to now, the question remains to what extent these results are generalizable. This cross-cultural study shows that in emerging countries, pain conditions can be treated with IOT and that treatment outcome may be equal or superior notwithstanding eventually less promising diagnoses being treated. Further, the study shows that not only treatment outcome in terms of pain reduction, but also perceived side effects are dependent on cultural factors to a considerable extent. Cross-cultural studies, not only on IOT but on pain therapy in general, can help to ensure the generalizability of findings related to the efficacy of specific therapies, and at the same time enhance our understanding of the cultural factors influencing the pain condition treated.
