# Correction to: A Cross-cultural Perspective on Intrathecal Opioid Therapy Between German and Iranian Patients

**DOI:** 10.1007/s11013-021-09753-2

**Published:** 2021-10-05

**Authors:** Barbara Kleinmann, Nayereh Khodashenas Firoozabadi, Tilman Wolter

**Affiliations:** 1 Interdisciplinary Pain Center, Medical Center, Faculty of Medicine, University of Freiburg, University of Freiburg, Breisacherstr. 64, 79106 Freiburg, Germany; 2 Department of Anesthesiology, Pain Clinic, Khatam Ol Anbia Hospital, Tehran, Islamic Republic of Iran

## Correction to: Cult Med Psychiatry (2021) 45:218–233 10.1007/s11013-020-09682-6

The article “A Cross-cultural Perspective on Intrathecal Opioid Therapy Between German and Iranian Patients”, written by Barbara Kleinmann ⋅ Nayereh Khodashenas Firoozabadi ⋅ Tilman Wolter was originally published electronically on the publisher’s internet portal on 28th July 2020 without open access. With the author(s)’ decision to opt for Open Choice the copyright of the article changed on 1st September 2021 to © The Author(s) 2021 and the article is forthwith distributed under a Creative Commons Attribution.

The original article has been corrected.

